# Mechanistic added value of a trans-Sulfonamide-Platinum-Complex in human melanoma cell lines and synergism with cis-Platin

**DOI:** 10.1186/s12943-017-0618-7

**Published:** 2017-02-23

**Authors:** Alba Agudo-López, Elena Prieto-García, José Alemán, Carlos Pérez, C. Vanesa Díaz-García, Lucía Parrilla-Rubio, Silvia Cabrera, Carmen Navarro-Ranninger, Hernán Cortés-Funes, José A. López-Martín, M. Teresa Agulló-Ortuño

**Affiliations:** 1 Laboratory of Translational Oncology, Instituto de Investigación Sanitaria Hospital 12 de Octubre (i + 12), Avda de Córdoba S/N, 28041 Madrid, Spain; 2 Organic Chemistry Department (Module 1), Universidad Autónoma de Madrid, C/Fco Tomás y Valiente, 5. Cantoblanco, 28049 Madrid, Spain; 3 Medical Oncology Service, Hospital Universitario 12 de Octubre, Avda de Córdoba S/N, 28041 Madrid, Spain; 4 Inorganic Chemistry Department (Module 7), Universidad Autónoma de Madrid, C/Fco Tomás y Valiente, 5, Cantoblanco, 28049 Madrid, Spain

**Keywords:** Cisplatin, Transplatin, Mono-Sulfonamide, Melanoma, Cell Cycle control, Mechanisms of action

## Abstract

**Background:**

Cisplatin is a potent antitumor agent. However, toxicity and primary and secondary resistance are major limitations of cisplatin-based chemotherapy, leading to therapeutic failure. We have previously reported that mono-sulfonamide platinum complexes have good antitumor activity against different tumoral cell lines and with a different and better cytotoxic profile than cisplatin. Besides, N-sulfonamides have been used extensively in medicinal chemistry as bactericides, anticonvulsant, inhibitors of the carbonic anhydrase, inhibitors of histone deacetylases, and inhibitors of microtubule polymerization, among others.

**Methods:**

We aimed to compare the cytotoxic effects of cisplatin and a *trans*-sulfonamide-platinum-complex (TSPC), in two human melanoma cell lines that differ in their TP53 status: SK-MEL-5, *TP53* wild type, and SK-MEL-28, *TP53* mutated. We performed cytotoxicity assays with both drugs, alone and in combination, cell cycle analyses, western blotting and immunoprecipitation, and fluorescence immunocytochemistry.

**Results:**

TSPC had similar antiproliferative activity than cisplatin against SK-MEL-5 (3.24 ± 1.08 vs 2.89 ± 1.12 μM) and higher against SK-MEL-28 cells (5.83 ± 1.06 vs 10.17 ± 1.29 μM). Combination of both drugs inhibited proliferation in both cell lines, being especially important in SK-MEL-28, and showing a synergistic effect. In contrast to cisplatin, TSPC caused G1 instead G2/M arrest in both cell lines. Our present findings indicate that the G1 arrest is associated with the induction of CDKN1A and CDKN1B proteins, and that this response is also present in melanoma cells containing *TP53* mutated. Also, strong accumulation of CDKN1A and CDKN1B in cells nuclei was seen upon TSPC treatment in both cell lines.

**Conclusions:**

Overall, these findings provide a new promising TSPC compound with in vitro antitumor activity against melanoma cell lines, and with a different mechanism of action from that of cisplatin. Besides, TSPC synergism with cisplatin facilitates its potential use for co-treatment to reduce toxicity and resistance against cisplatin. TSPC remains a promising lead compound for the generation of novel antineoplastic agent and to explore its synergism with other DNA damaging agents.

## Background

Cisplatin (*cis*-diamminedichloroplatinum(II) or CDDP) is one of the most successful traditional antitumoral metal compounds used in oncology. Its mode of action is mediated by its interaction with DNA to form DNA adducts, which activate several signal transduction pathways leading to cell death [1, 2]. However, it is associated with different adverse side effects, such as dose-limiting nephrotoxicity, peripheral neuropathy, electrolyte disturbance, tinnitus and hearing loss [3]. In addition, primary or secondary resistance to CDDP may occur, leading to therapeutic failure [2, 4]. Many platinum compounds have been designed with the aim to broaden the spectrum of activity, reduce side effects, and overcome resistance to CDDP [5–8].

On the other hand, *N-*sulfonamides have been used extensively in medicinal chemistry as inhibitors of histone deacetylases and microtubule polymerization, among other targets [9–11]. Besides the large number of publications concerning to the use of sulfonamides, the synthesis of platinum compounds containing sulfonamides in their structure has been scarce. The most promising results are given by those compounds which activate alternative signaling pathways [12, 13]. Therefore, research on new metallodrugs remains an area of interest, especially those with different mechanisms of action from CDDP.

**Fig. 1:**
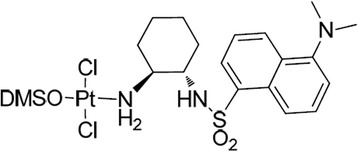
*trans*-Dichlorido [(*rac*)-2-(5-(dimethylamino)naphthalene-1-sulfonamido)cyclohexylamino] (dimethylsulfoxide)platinum(II) compound (TSPC). Chemical Formula: C_20_H_31_Cl_2_N_3_O_3_PtS_2_ Molecular Weight: 691.5924 g/mol

Recently, we published the synthesis and evaluation of a series of *trans*-sulfonamide platinum complexes showing antitumor activity [14]. Among these compounds, *trans*-Dichlorido [(*rac*)-2-(5-(dimethylamino)naphthalene-1-sulfonamido)cyclohexylamino] (dimethylsulfoxide)platinum(II) (hereinafter TSPC), (Fig. 1) demonstrated similar or superior activity than CDDP against a panel of tumor cell lines, including melanoma. Melanoma is a particularly aggressive cancer that has poor prognosis due to resistance to multiple chemotherapy regimens, including in most cases CDDP [15, 16]. This highlights the urgency of implementing treatment strategies for melanoma with more effective and less toxic novel drugs.

Therefore, we decided to study several mechanistics aspects of TSPC in two melanoma cell lines with a different TP53 status: SK-MEL-5 (*TP53* wild-type) and SK-MEL-28 (*TP53* mutated) [17]. TP53 is a tumor suppressor protein that facilitates antitumor drug response using a variety of key cellular functions, including cell cycle arrest, senescence, and apoptosis [18], whose role in the mode of action of CDDP has been extensively described [1]. These functions usually cease once TP53 mutates, as occurs in nearly 50% of cancers, and some TP53 mutants even exhibit gain-of-function effects, which can lead to even greater drug resistance [18]. It is therefore important to test the effectiveness of new drugs in TP53 mutants.

In this study, we show the in vitro antitumor activity of TSPC and CDDP in these melanoma cell lines and mechanistic differences in cell cycle effects. We also aim to explore if these differential effects might be used as a basis for a synergism between both compounds.

## Methods

### Cell lines and reagents

Human melanoma cell lines SK-MEL-5 and SK-MEL-28 were obtained from American Type Culture Collection (ATCC, Manassas, VA) in February 2010. Low passage cells were cultured in RPMI 1640 medium supplemented with 10% fetal bovine serum, 100 U/mL penicillin, 100 μg/mL streptomycin and 2 mM L-glutamine, under standard culture conditions (37 °C, 95% humidified air and 5% CO_2_). RPMI 1640 and other culture materials were from Lonza Ltd. (Verviers, BEL). TSPC was synthesized and characterized in the Inorganic Chemistry Department of the Universidad Autónoma de Madrid (Spain), as previously reported [14]. CDDP was obtained from Selleck Chemicals LLC (Houston, TX). Drugs were dissolved in dimethyl sulfoxide (DMSO) at 100 mM of stock solution, and stored at −20 °C until use.

### Proliferation assays

Briefly, cells were seeded in a 96-well flat-bottom plate at 5000 cells/well and cultured for 24 h prior to exposure to CDDP or TSPC at varying concentrations, from 0 to 100 μM for 72 h. For combination assays, cells were treated with CDDP ranging from 0.5 to 10 μM and two different doses of TSPC (1 and 5 μM). Results were expressed as a percentage relative to vehicle-treated control (0.1% DMSO was added to untreated cells). Viability was determined using the WST-1 method (Roche, Mannheim) following the manufacter’s instructions. The 50% inhibitory concentration (IC_50_) was calculated by nonlinear regression fit of the mean values of the data obtained in at least three independent experiments using GraphPad Prism software version 5.0 (San Diego, CA).

The type of drug interaction was analysed by the Chou-Talalay method [19]. This method provides a combination index (CI) that allows quantitative determination of drug interactions, where CI < 1, 1, and >1 indicate synergism, additive effect or antagonism, respectively, considering synergism as more than additive effect and antagonism as less than additive effect. This method also provides a dose reduction index (DRI) that measure how many-fold the dose of a drug can be reduced in combination with respect to the drug alone. The CI and the DRI were calculated using the CompuSyn software (Comb ComboSyn Inc, Paramus, NJ).

### Cell cycle and apoptosis analysis

Cells were treated for 24 h with equimolar concentrations of either CDDP or TSPC. The cell cycle progression was examined by flow cytometry after staining with propidium iodide (PI). DNA content and cell cycle analyses were performed by using a FACScalibur flow cytometer and the CellQuest software (Becton Dickinson Biosciences). Apoptosis was studied with the APO-BrdUTunel assay kit (Life Technologies Inc. Gaithersburg, MD) following the experimental protocol provided by the manufacturer, and analyzed with a FACScalibur flow cytometer and the CellQuest software (BD Biosciences). All experiments were performed in triplicate.

### Western blot and immunoprecipitation assays

Cells untreated (control) or treated with equimolar concentrations of either CDDP or TSPC for 24 h were lysed with MCL1 lysis buffer in the presence of protease and phosphatase cocktail inhibitor (Sigma-Aldrich Co. LLC, St Louis, MO) following the manufacter’s protocol. Total protein concentrations were determined using the BCA protein assay kit (Thermo Scientific Meridian Rd, Rockford, IL). Protein lysates (30 μg) were subjected to SDS-PAGE on 15% polyacrylamide gel. The separated proteins were transferred on to PVDF membrane followed by blocking with 5% non-fat milk powder (w/v) in TTBS (10 mM Tris, 100 mM NaCl, 0.1% Tween 20). Membranes were probed for the protein levels of TP53, CDKN1A (p21^Cip1^), CDKN1B (p27^Kip1^), CDKN2B (p15^INK4b^), CDK2, CDK4, CCND1 (Cyclin D1), CCNE2 (Cyclin E2), phospho-CDK1 (P-cdc2) and GAPDH using specific primary antibodies (Cell Signaling Tech Inc, Danvers, MA) diluted 1/1000 in blocking solution followed by peroxidase-conjugated appropriate secondary antibody (Santa Cruz Biotechnology Inc, Dallas, TX), and visualized by Immun-Star WesternC kit (Bio-Rad) detection system. Results were scanned with Image Quant LAS 4000 Imaging densitometer and quantify with Image Quant TL software (GE healthcare, Life Sciences). To ensure that control cells gave reproducible baseline protein levels, we carefully maintained cells cultures in a constant semiconfluent and logarithmically growing state throughout each experiment.

Pierce Classic IP Kit (Thermo Scientific) was used for immunoprecipitation assays, and manufacturer’s instructions were followed. Thereby, 1 mg of protein was immunoprecipitated using CDK2 primary antibody (Cell Signaling Tech Inc, Danvers, MA). The immune complex eluted was subjected to SDS-PAGE and trans-blotted into PVDF membranes as described above. Membranes were probed for the protein levels of CDK2, CDKN1A and CDKN1B.

### Fluorescence immunocytochemistry

Cells were cultured in coverslip glasses for 24 h with equimolar concentration of either CDDP or TSPC. Untreated cells were used as control. After exposure to the drugs, cells were fixed with 4% formaldehyde and permeabilized with PBS/triton (0.1%). For immunofluorescence labeling, cells were incubated with anti-human CDKN1A (1:1000) or CDKN1B (1:1000) monoclonal antibodies (Cell Signaling Tech Inc, Danvers, MA) followed by a secondary antibody conjugated to Alexa Fluor 488 (Life Technologies Inc; Gaithersburg, MD). Nuclei were stained with 2-(4-amidinophenyl)-1H-indole-6-carboxamidine (DAPI) (Life Technologies Inc. Gaithersburg, MD). Images from six to ten fields per sample of at least three independent experiments were taken with a LSM 510 Meta Confocal Microscope (Zeiss, Germany).

### Recovery experiments

To study the effects of duration of platinum exposure on cell proliferation, cells were treated with CDDP or TSPC at their corresponding IC_50_ for 72 h, or with CDDP 1 μM (SK-MEL-5) or 3 μM (SK-MEL-28) and TSPC 1 or 5 μM in combination assays. After this time, cells were washed with PBS and put in drug-free medium to recover. Viable cell number was assessed with Trypan blue and a Neubauer chamber for 7 days of recovery.

### Statistical analysis

Results are expressed as the mean ± SD, and data are representative of at least three different experiments. Analysis of variance (ANOVA) one way, followed by Dunnett’s *t*-test was used to examine differences between groups. For recovery experiments, cell growth was fit to a Gompertz function, typical of cell growth and characterized by a lag period, an exponential and a saturation phase. All the statistical tests were conducted at the two-side 0.05 level of significance.

## Results

### TSPC inhibits growth and induces G1 arrest in human melanoma SK-MEL-5 and SK-MEL-28 cells

**Fig. 2:**
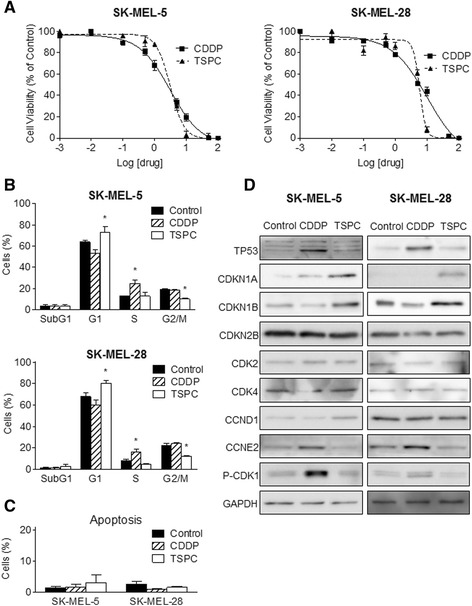
Effect of CDDP and TSPC on melanoma cell lines. **a** Log-dose response curves for SK-MEL-5 and SK-MEL-28 cells following treatment with CDDP or TSPC at 72 h. **b** Distribution of cells between cell cycle phases after CDDP and TSPC exposure. (*, *p* < 0.05). **c** Apoptotic effect of CDDP and TSPC evaluated by APO-BrDUTunel assay. The experiments were carried out in triplicate. The results are shown as the mean ± standard deviation. **d** Effect of CDDP and TSPC in cell cycle regulatory proteins TP53, CDKN1A, CDKN1B, CDKN2B, CDK2, CDK4, CCND1 (cyclin D1), CCNE2 (cyclin E2), and P-CDK1 as determined by Western blot analysis. GAPDH is shown as loading control

Based on our promising previous work with TSPC [14] we decided to study its effect in two melanoma cell lines: SK-MEL-5 (*TP53* wild-type) and SK-MEL-28 (*TP53* mutated). First, we analysed TSPC antiproliferative activity. As shown in Fig. 2a, TSPC treatment inhibited the growth of human melanoma SK-MEL-5 and SK-MEL-28 cells in a dose-dependent manner. The IC_50_ of this compound (3.24 ± 1.08 μM) was similar to the one obtained with CDDP (2.89 ± 1.12 μM) in SK-MEL-5, and significantly lower (*p* < 0.05) in SK-MEL-28 (5.83 ± 1.06 μM vs 10.17 ± 1.29 μM).

Next, we examined its effect on cell cycle progression and in apoptosis induction. Consistent with the results on cell growth inhibition, TSPC treatment for 24 h induced a significant (*p* < 0.05) increment of cell number in G1 phase in both melanoma cell lines (Fig. 2b), whereas CDDP treatment induced S arrest, as has been previously described elsewhere [2]. Apoptosis was not observed by TUNEL assay after 24 h treatment with neither CDDP nor TSPC, which is consistent with the subG1 cell population identified in cell cycle analyses (Figs. 2b and c). These effects were apparent in both cell lines after a 24-h exposure to any of the two compounds.

### Effect of TSPC on G1 cell cycle regulators

Based on the G1 arrest induced by TSPC in both cell lines, we assessed the effect of this compound on the cell cycle regulatory molecules that play important roles in G1 phase of cell cycle progression. TP53 is the most important tumor suppressor protein associated with the G0-G1 arrest in cell cycle, and a key protein in CDDP mode of action and resistance. Since SK-MEL-28 has a mutation in *TP53*, we also examined the protein expression of the cyclin-CDK inhibitors (CKIs) CDKN1A (p21^Cip1^), CDKN1B (p27^Kip1^), and CDKN2B (p15^INK4b^).

Western blot analysis showed that TSPC treatment strongly increased the protein levels of CDKN1A and CDKN1B and had no effect on TP53 levels in both cell lines (Fig. 2d). The observed strong induction in CDKN1A and CDKN1B protein levels by TSPC was not due to an overall change in protein levels as confirmed by probing the same membranes with GAPDH antibody (Fig. 2d). Thus, treatment of SK-MEL-5 and SK-MEL-28 cells with TSPC caused an increased in CDKN1A and CDKN1B expression, with correlated with the G1 arrest at 24 h. Conversely, CDDP increased the protein levels of TP53 in both cell lines accompanied by an increase in CDKN1A in SK-MEL-5 but not in SK-MEL-28. CDDP also slightly decreased the protein levels of CDKN1B in both cell lines. No differences were observed in CDKN2B levels with any treatment.

We also assessed the effect of TSPC on the protein levels of CDKs and cyclins involved in G1 phase and G1 to S phase transition of cell cycle. As shown in Fig. 2d, drug treatments did not show any detectable change in the protein levels of CDK2 and CDK4, and only a slight increase in cyclin D1 with TSPC in SK-MEL-5 was observed. On the other hand, cyclin E2 levels were strongly increased by CDDP, and conversely reduced by TSPC in both cell lines.

**Fig. 3:**
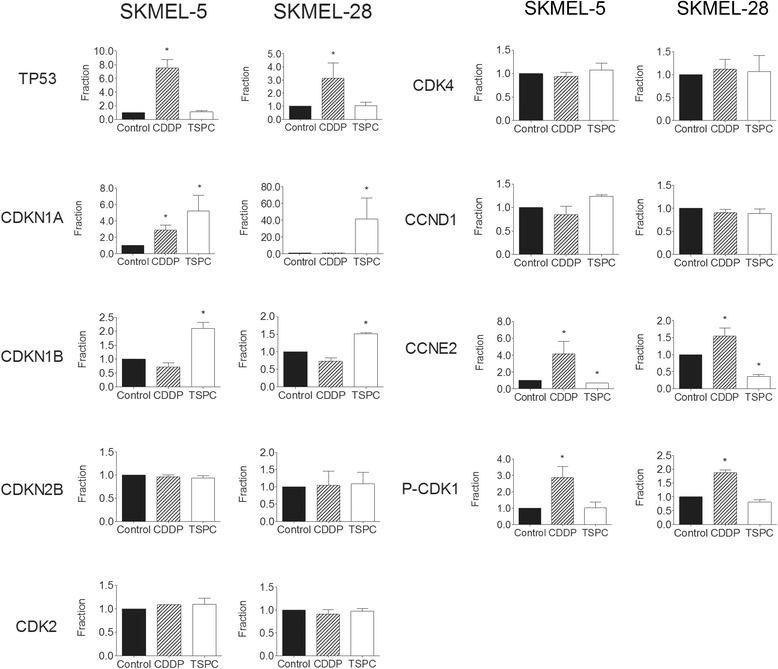
Quantification of effect of CDDP and TSPC in cell cycle regulatory proteins TP53, CDKN1A, CDKN1B, CDKN2B, CDK2, CDK4, CCND1, CCNE2, and P-CDK1 as determined by Western blot analysis of three independent experiments

In order to assess the mechanism of action of CDDP in the absence of TP53, we also examined the phosphorylation state of CDK1 (cdc2), which have been shown to be associated with S and G2/M arrest and with CDDP mechanism of action. Western blot analysis showed that CDDP treatment strongly induced the protein levels of phospho-CDK1 in both cell lines, while TSPC had no effect (Fig. 2d). Densitometric analysis of proteins from Fig. 2d is show in Fig. 3.

### TSPC inhibits CDK2-cyclin E via CDKN1A and CDKN1B

**Fig. 4:**
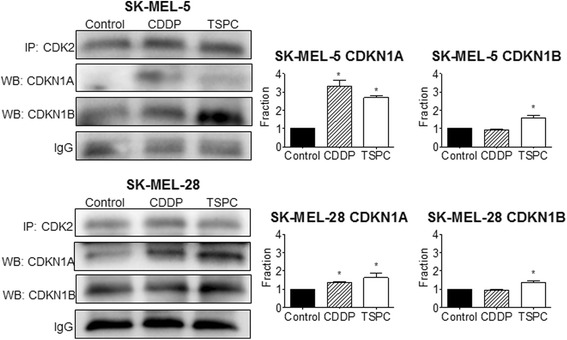
Co-immunoprecipitation assay of the interaction between CDK2 and CDKN1A, and CDK2 and CDKN1B in SK-MEL-5 and SK-MEL-28 cell lines after CDDP and TSPC exposure. Quantification of CDKN1A and CDKN1B binds to CDK2, detected in co-immunoprecipitated products. (*, *p* < 0.05)

Since up-regulation in CKIs levels was observed, and these proteins exert their inhibitory effect and G1 cell cycle arrest by direct binding to CDK2-cyclin E complexes, we next examined CDK2-CKIs binding by immunoprecipitation assay. As shown Fig. 4, TSPC treatment showed an increased binding of CDKN1A and CDKN1B with CDK2 in both cell lines. CDDP also showed an increased binding of CDKN1A, bigger than that observed with TSPC in SK-MEL-5.

**Fig. 5:**
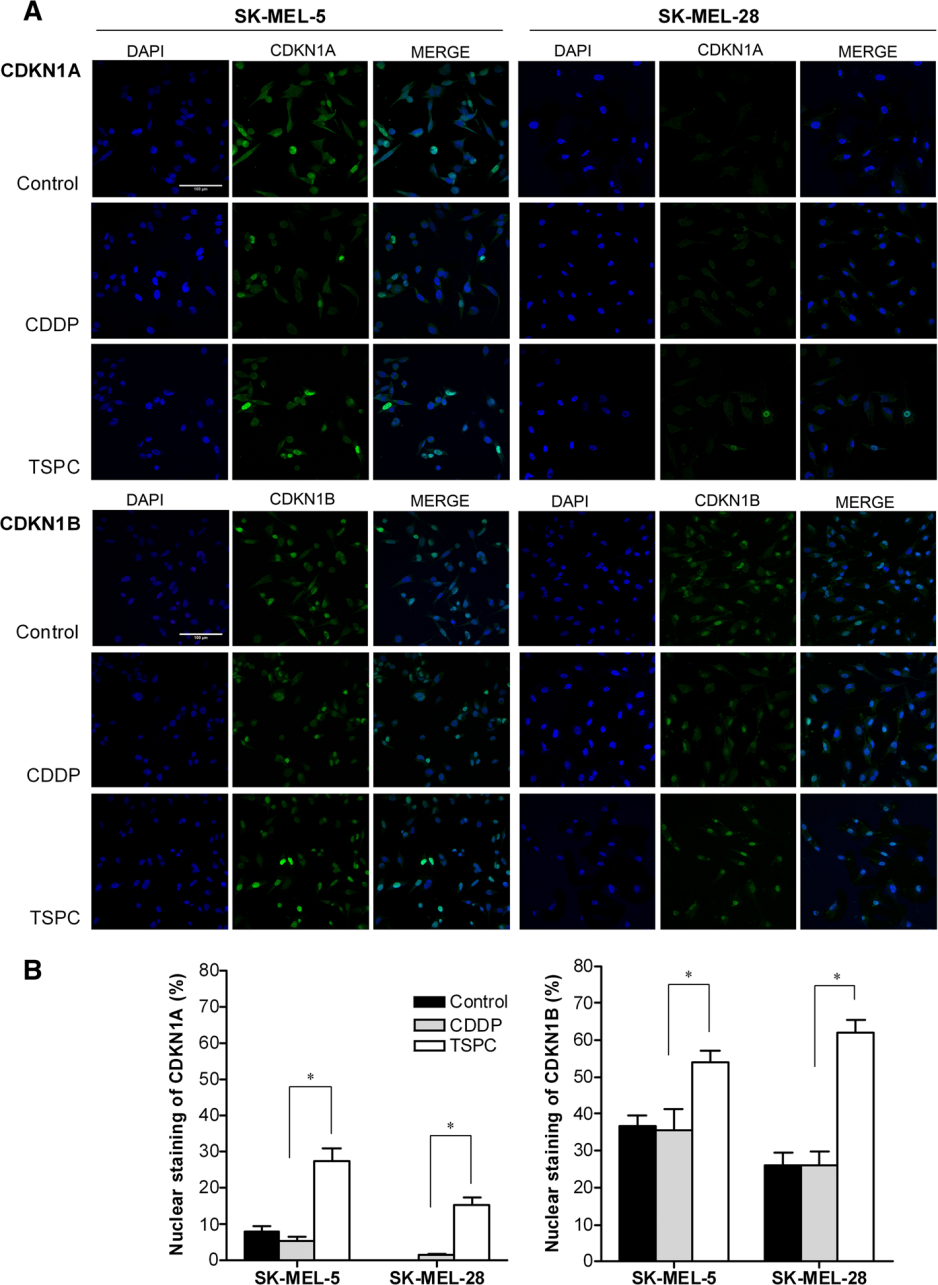
Cellular distribution of CDKN1A and CDKN1B in melanoma cell lines and its alteration after CDDP or TSPC treatment. **a** Subcellular distribution of CDKN1A and CDKN1B in SK-MEL-5 and SK-MEL-28 cells, after CDDP and TSPC treatment assessed by confocal microscopy. First and fourth columns: nuclei stained with DAPI; Second and fifth columns: CDKN1A (*up*) and CDKN1B (*down*) proteins stained with specific antibody and secondary antibody conjugated with Alexa Fluor 488; Third and sixth columns: colocalization. **b** Quantitative analysis of nuclear CDKN1A (*left*) and CDKN1B (*right*) staining in melanoma cell lines. Staining cells were counted at 200 × magnification from six to ten randomly selected fields. Total 100 cells were counted in each experiment. (*, *p* < 0.05)

The inhibitory effect of CKIs on CDK-cyclin complexes occurs in the nucleus, so we next examined by immunofluorescence and confocal microscopy the localization of CKIs in this cell lines. As shown in Fig. 5a, both CDKN1A and CDKN1B were mainly localized in the nucleus in untreated cells. TSPC exposure induced an increase of CDKN1A and CDKN1B in the nucleus in SK-MEL-5 and SK-MEL-28, while CDDP treatment seemed to slightly modified CDKN1B levels in both cell lines. No CDKN1A staining was perceivable in SK-MEL-28 in control and CDDP-treated cells. Confocal images matched the results obtained by western blot (Fig. 2d).

In addition, we determined the percentage of nuclear CDKN1A and CDKN1B staining in cell lines exposed to CDDP or TSPC. As shown in Fig. 5b, cells treated with TSPC had a higher percentage of nuclear CDKN1A and CDKN1B than cells treated with CDDP (SK-MEL-5: CDKN1A 25.36 ± 11.53 vs 5.45 ± 4.16; CDKN1B 54.04 ± 10.36 vs 30.70 ± 8.85) (SK-MEL-28: CDKN1A 15.31 ± 5.28 vs 1.5 ± 0.63; CDKN1B 62.11 ± 10.50 vs 23.53 ± 8.31). No significant changes of CDKN1A and CDKN1B subcellular location were observed after treatment with CDDP. By contrast, a significant increase in the nuclear staining of both CKIs after exposure to TSPC was seen.

### TSPC in combination

**Fig. 6:**
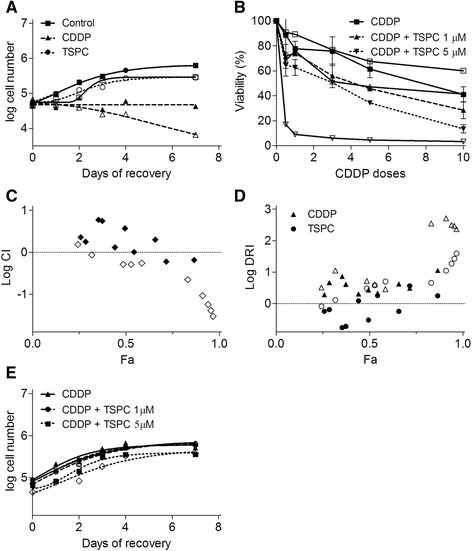
Effects of CDDP and TSPC combined treatment. **a** Cell recovery after treatment removal. Cell growth was assessed for 7 days in cells treated with either CDDP (*triangles*) or TSPC (*circles*) at their corresponding IC_50_ for 72 h followed by drug removal. Untreated cells were used as control (*squares*). **b** Cell viability with drug treatment combination. Viability after treatment with different doses of CDDP ranging from 0.5 to 10 μM combined with TSPC 1 μM (*right side-up triangles*) or 5 μM (*upside-down triangles*). Cells just treated with CDDP were used as control (*squares*). **c** Fa-log CI plot. The line represents the additive effect and all the points under it show synergism. **d** Fa-log DRI plot. The line separates the favourable dose reduction (*up*) from the unfavourable (*down*). **e** Cell recovery after combination treatment removal. Clear shape symbol: SK-MEL-5; Dark shape symbol: SK-MEL-28

Once analysed the effect of TSPC on cell cycle and cell growth, we next examined how long this effect would last. Cell treated with TSPC recovered a normal proliferation rate after drug removal. However, cell treated with CDDP did not recover (Fig. 6a). Seeing these results, as well as the differential effects in cell cycle, we thought it could be interesting to explore evidences for synergism using both drugs in combination. As shown in Fig. 6b, addition of TSPC to CDDP increased cell death in a dose dependent manner in both cell lines, being especially important in the case of SK-MEL-28 with TSPC 5 μM. In fact, the analysis of these drugs interaction by the Chou-Talalay method showed that this increment in cell death was due to a synergistic effect between the drugs (CI < 1; logCI < 0) in SK-MEL-28 at almost every dose and, in SK-MEL-5 at the highest doses of CDDP (Fig. 6c). Due to this synergism, there was a favourable DRI (DRI > 1; logDRI > 0) in CDDP dose and in some doses of TSPC in SK-MEL-5 and in both CDDP and TSPC in SK-MEL-28 (Fig. 6d).

Taking into consideration this synergism we studied whether cells treated with both drugs in combination were able to recover after drug removal. For this purpose we treated cells with CDDP at a dose lower than their respective IC_50_: 1 μM (SK-MEL-5) or 3 μM (SK-MEL-28), and TSPC 1 or 5 μM. In all the cases cells recovered in the same way (Fig. 6e).

## Discussion

Platinum complexes are widely used in cancer therapy. The successful clinical applications of CDDP have inspired the synthesis and investigation of numerous platinum compounds as drug candidates. All the commercially available platinum compounds are based in a *cis-*isomerism.

In this study, the cellular and molecular effects of the *trans*-platinum derivative TSPC were investigated and directly compared with those of CDDP in SK-MEL-5 and SK-MEL-28 melanoma cell lines. The choice of these cell lines were made on the basis of TP53 status, and in our previous cytotoxicity results in a panel of different cell lines [14].

TSPC showed to be as active as CDDP in SK-MEL-5 and more active in SK-MEL-28, the *TP53* mutant line. TP53 plays a very important role in CDDP cytotoxicity [20], so it is not surprising that a *TP53* mutant line show some resistance to this drug [21, 22]. On the other hand, and according to the results obtained in this work, TSPC mode of action is clearly TP53-independent, as there is no change in this protein levels after TSPC treatment, neither in SK-MEL-5 nor in SK-MEL-28 cell lines.

**Fig. 7:**
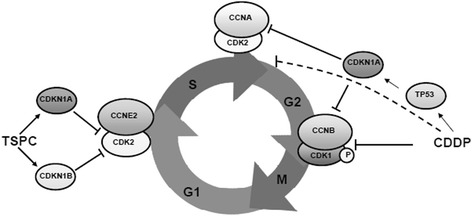
TSPC induces cell cycle G1 arrest probably mediated by CDKN1A and CDKN1B CKIs in a TP53-independent manner, while CDDP induces S and G2/M arrest via TP53/CDKN1A pathway in SK-MEL-5, and preventing dephosphorylation of CDK1 in both cell lines

Upon 24 h incubation with TSPC, cells were arrested in G1 phase that suggest cell cycle delay for a DNA damage response, DNA repair and/or apoptosis. One goal of this study was to define the cell cycle regulatory pathway responsible for the G1 arrest. The results obtained in these two cell lines with different TP53 status show that TSPC inhibits cell proliferation through cell cycle arrest at G1 phase, likely due to the up-regulation of CDK inhibitors CDKN1A and CDKN1B. Progression through the G1 phase of the cell cycle is controlled by CDK4-cyclin D complexes, while CDK2-cyclin E complexes are required for proper G1/S transition and initiation of S [23]. CDKs are negatively regulated by the binding of CDK inhibitors. INK4 family inhibitors, as CDKN2B, prevent cyclin D binding to CDK4, while CDKN1A and CDKN1B bind to CDK2-cyclin E complexes and inhibit CDK2 activity [24]. Our results showed that neither CDK4, nor cyclin D1, nor CDKN2B were affected by TSPC in SK-MEL-28 cells. Only a slight increase in cyclin D1 was observed in SK-MEL-5 cells but such levels might be insufficient to induce G1 progression effectively. However, CDKN1A and CDKN1B protein levels were clearly enhanced after TSPC treatment, and cyclin E2 levels were reduced in both cell lines. Besides, co-immunoprecipitation assays showed that both CDKN1A and CDKN1B bind to CDK2 after TSPC treatment. Thus, it can be inferred that TSPC induces G1 arrest in SK-MEL-5 and SK-MEL-28, via CDKN1A and CDKN1B binding to CDK2-cyclin E complexes in a TP53-independent manner (Fig. 7). The cell cycle arrest is a common cellular response to DNA damage and is viewed as a delay period in DNA replication during which the cell can attempt to repair the damage. If this attempt fails, cell death pathways will be activated [25, 26]. Our present findings indicate that the G1 arrest is associated with an induction of CDKN1A and CDKN1B proteins, and that this response is also seen in melanoma cells containing TP53 mutated.

On the other hand, cells incubated with CDDP were arrested in S and G2/M phase. Several reports have previously described that CDDP induces S- and G2/M-phase arrests in a sequential manner [27]. These arrests are associated with phosphorylation and proteosomal degradation of CDC25 phosphatase, with the consequence that CDK within the CDK2/cyclin A and CDK1/cyclin B complexes remain in the inhibitory tyrosine phosphorylated state [28]. Besides, CDKN1A can bind these CDKs and inhibit these complexes [29], and as an inhibitor of CDC2 phosphorylation, CDKN1A can be involved in both G1 and G2 cell cycle arrest [30]. Our data from the present study are in line with these previously published studies as CDK1 remain phosphorylated after CDDP treatment, and CDKN1A binds to and inhibit CDK2. However, this setting is different to that of TSPC, where CDC2 remains hypophosphorylated. So, it seems that TSPC has a different mode of action from that of CDDP.

The cell cycle regulatory function of both CDKN1A and CDKN1B is associated with its nuclear localization, but the protein can also localize in the cytoplasm where they could exert different functions [31]. In the nucleus, in addition to the regulation of the cell cycle progression, CDKN1A and CDKN1B are also involved in a variety of transcriptional responses [32]. In the cytoplasm, CDKN1A can initiate antiapoptotic responses by inhibiting proapoptotic kinase ASK1 [33] or by binding to procaspase 3, thus preventing its proteolytic activation [34]. In a more general sense, it has been suggested that the subcellular localization of CDKN1A defines its function as a tumor suppressor (nuclear localization) or oncoprotein (cytoplasmic localization) [32]. Indeed, some human cancers display elevated levels of cytoplasmic CDKN1A or cytoplasmic CDKN1B, which is associated with poor prognosis [35–38], and badly response to cisplatin based treatment [39, 40]. In our experiments, the increased nuclear localization of CDKN1A and CDKN1B in both melanoma cell lines after TSPC treatment supports the antitumor activity of this compound.

Recovery experiments showed that while CDDP effect, at least at IC_50_ dose, was permanent, TSPC treatment was transitory. This is not necessarily a negative result, but it implies that TSPC treatment should be frequent. Thus, in regard to clinical treatment, it would be useful to find an easy and sequentially way of TSPC administration.

CDDP is one of the most potent antitumor agents known. However, toxicity and resistance are major limitations of CDDP-based chemotherapy [1, 2]. Thus, the potential for use of TSPC in combination with CDDP was explored in terms of eventual synergism. In both cell lines tested, our data show a synergistic effect and a favorable DRI when cells are simultaneously treated with both CDDP and TSPC, which could lead to resistance minimization. This synergy is likely to be due to the mechanistic differences between CDDP and TSPC.

## Conclusions

In this study we present a new promising TSPC compound with in vitro antitumor activity against SK-MEL-5 and SK-MEL-28 melanoma cell lines, with a different mechanism of action that of CDDP. From our results it can be inferred that TSPC induces cell cycle G1 arrest probably mediated by CDKN1A and CDKN1B inhibitors in a TP53-independent manner. CDK2-cyclin E complexes are required for G1/S transition. By co-immunoprecipitation assays, we show that CDKN1A and CDKN1B bind to CDK2 after TSPC treatment, inhibiting CDK2 activity. Besides, synergy between TSPC and CDDP facilitates its potential use for co-treatment to reduce toxicity and resistance against CDDP. On the other hand, TSPC remains a promising lead compound for the generation of novel drug candidates with different cytotoxicity profiles from those of CDDP. Moreover, these results could have therapeutic implications for TSPC, because the majority of human solid tumors contain a mutant TP-53 or deleted *TP53* gene and are inherently resistant to commonly used DNA-damaging agents.

## Abbreviations

CDDP: Cisplatin or *cis*-diamminedichloroplatinum(II)
CI: Combination index
CKIs: Cyclin-CDK inhibitors
DRI: Dose reduction index
TSPC: *Trans*-sulfonamide-platinum-complex
